# Genotyping of *Coxiella burnetii* from domestic ruminants in northern Spain

**DOI:** 10.1186/1746-6148-8-241

**Published:** 2012-12-10

**Authors:** Ianire Astobiza, Jeroen JHC Tilburg, Alvaro Piñero, Ana Hurtado, Ana L García-Pérez, Marrigje H Nabuurs-Franssen, Corné HW Klaassen

**Affiliations:** 1 NEIKER-Instituto Vasco de Investigación y Desarrollo Agrario, Department of Animal Health, Derio, Bizkaia, Spain; 2 Canisius Wilhelmina Hospital, Department of Medical Microbiology & Infectious Diseases, Nijmegen, The Netherlands

**Keywords:** Q fever, Ruminants, Genotyping, MLVA, MST, Spain

## Abstract

**Background:**

Information on the genotypic diversity of *Coxiella burnetii* isolates from infected domestic ruminants in Spain is limited. The aim of this study was to identify the *C. burnetii* genotypes infecting livestock in Northern Spain and compare them to other European genotypes. A commercial real-time PCR targeting the IS*1111a* insertion element was used to detect the presence of *C. burnetii* DNA in domestic ruminants from Spain. Genotypes were determined by a 6-loci Multiple Locus Variable number tandem repeat analysis (MLVA) panel and Multispacer Sequence Typing (MST).

**Results:**

A total of 45 samples from 4 goat herds (placentas, N = 4), 12 dairy cattle herds (vaginal mucus, individual milk, bulk tank milk, aerosols, N = 20) and 5 sheep flocks (placenta, vaginal swabs, faeces, air samples, dust, N = 21) were included in the study. Samples from goats and sheep were obtained from herds which had suffered abortions suspected to be caused by *C. burnetii*, whereas cattle samples were obtained from animals with reproductive problems compatible with *C. burnetii* infection, or consisted of bulk tank milk (BTM) samples from a Q fever surveillance programme. *C. burnetii* genotypes identified in ruminants from Spain were compared to those detected in other countries. Three MLVA genotypes were found in 4 goat farms, 7 MLVA genotypes were identified in 12 cattle herds and 4 MLVA genotypes were identified in 5 sheep flocks. Clustering of the MLVA genotypes using the minimum spanning tree method showed a high degree of genetic similarity between most MLVA genotypes. Overall 11 different MLVA genotypes were obtained corresponding to 4 different MST genotypes: MST genotype 13, identified in goat, sheep and cattle from Spain; MST genotype 18, only identified in goats; and, MST genotypes 8 and 20, identified in small ruminants and cattle, respectively. All these genotypes had been previously identified in animal and human clinical samples from several European countries, but some of the MLVA genotypes are described here for the first time.

**Conclusions:**

Genotyping revealed a substantial genetic diversity among domestic ruminants from Northern Spain.

## Background

*Coxiella burnetii* is ubiquitous and the causative agent of Q fever, a zoonotic disease [1]. Domestic ruminants are often asymptomatic carriers of *C. burnetii* and are considered the most important reservoir and source for human Q fever infection [2]. However, other animal species like birds, reptiles, arthropods or pets can also be infected and possibly transmit the disease to humans [1].

*C. burnetii* can cause abortions and stillbirths in goats and sheep, and infertility and endometritis in cattle [3]. Infected animals shed bacteria mainly through milk, faeces, vaginal mucus and birth products [4]. Inhalation of *C. burnetii* contaminated aerosols is the main route of infection for humans. *C. burnetii* can be transported by the wind several kilometres far from the original infected source; direct contact with animals or *C. burnetii* infected birth products is not always necessary [5].

Considering the impact of *C. burnetii* on human and animal health, the study of potential sources of infection and the characterization of strains present in an area is of great epidemiological importance. Genotypic characterization of *Coxiella burnetii* is a prerequisite for surveillance purposes and for epidemiological investigation of Q fever outbreaks. This information is necessary to evaluate the epidemiological link between the source of the outbreak and human cases, with the final objective of establishing control measures in potential animal hosts involved in the life cycle.

Several techniques have been used to genotype and characterize *C. burnetii* strains. Techniques such as pulsed field gel electrophoresis were able to classify *C. burnetii* isolates in different groups [6]. DNA restriction fingerprints and separation by SDS-PAGE differentiated six genomic groups [7]. The analysis of the sequences of certain genes such as *com*1, *icd* or *muc*Z has been used for differentiating *C. burnetii* isolates [8-10]. More recently, multiple locus variable number tandem repeats analysis (MLVA) [11-15] and multispacer sequence typing (MST) [16,17] proved to be reliable techniques, reproducible, and with a high discriminatory power. In addition, these techniques do not require previous cultivation of the bacteria which is very difficult and requires biosafety level 3 conditions, and can be implemented directly on clinical and/or environmental samples.

Q fever is an endemic disease in ruminants in several regions of Spain. Recent seroprevalence studies carried out in Northern, Central and Southern Spain revealed the importance of domestic ruminants as reservoir for this zoonosis [18-20], with herd seroprevalence ranging between 30% and 75% depending on the ruminant species, and individual seroprevalence ranging between 6% and 60%. However, information on the genotypic diversity of *C. burnetii* isolates from domestic ruminants in Spain is limited [21]. The aim of this study was to identify the *C. burnetii* MLVA and MST genotypes that infect livestock in Spain and to compare them to other European genotypes.

## Methods

### Samples

A total of 45 samples from 4 goat herds (N = 4), 12 dairy cattle herds (N = 20) and 5 sheep flocks (N = 21) were included in the study. Details on the geographic origin of the samples, year of collection, type of reproductive disorders at the time of sampling, and number and type of samples collected in each farm are shown in Table 1. Ovine and caprine samples were collected for laboratory diagnosis by clinical veterinarians as part of the usual clinical practice on farms with abortions or reproductive problems, and Spanish ethical guidelines (RD 1201/2005) and animal welfare regulations were strictly respected. All herd owners had given an informed consent prior to the study. Samples from cattle farms were collected within a research project on Q fever in dairy cattle farms and experimental work was officially approved by competent local authorities (Diputación Foral de Bizkaia, reference 10559, 3rd November 2010). Environmental samples consisted on aerosol samples and dust taken from animal premises. Air was sampled using a Sartorius air sampler (Air Sampler, MD8 airscan, Goettingen, Germany) at a flow rate of 100 l/min for 10 min. and particles were collected in gelatine filters which were processed for DNA extraction.

**Table 1:** **Description of material examined for *C. burnetii *genotyping**

**Farm**^**1**^	**Location**^**2**^	**Year**	**Reproductive disorders**	**Sample type**
Gt1	AL	2010	Abortion	1 Placenta
Gt2	BI	2010	Abortion	1 Placenta
Gt3	TO	2010	Abortion	1 Placenta
Gt4	ZA	2005	Abortion	1 Placenta
DC1	GI	2011	Infertility, abortion	3 vaginal mucus, 2 milk, 1 aerosol
DC2	BI	2010	Infertility	1 BTM, 2 milk
DC3	BI	2010	Infertility	2 individual milk
DC4	BI	2011	Abortion, infertility	1 vaginal mucus
DC5	NA	2011	Infertility	1 individual milk
DC6	CA	2011	Metritis, infertility	1 BTM
DC7	LU	2011	Abortion, infertility	1 individual milk
DC8	BI	2009	No	1 BTM
DC9	BI	2009	No	1 BTM
DC10	BI	2010	No	1 BTM
DC11	BI	2009	No	1 BTM
DC12	BI	2009	Metritis	1 BTM
Sh1	AL	2004	Abortion	1 Placenta
Sh2	SS	2007-09	Abortion	1 vaginal mucus, 1 individual milk, 2 faeces, 2 aerosols
Sh3	SS	2008-11	Abortion	3 vaginal mucus, 1 placenta, 1 aerosol
Sh4	SS	2008-11	Abortion	2 vaginal mucus, 1 aerosol, 2 dust samples
Sh5	SS	2008-11	Abortion	1 vaginal mucus, 1 faeces, 1 aerosol, 1 dust sample

^1^ Farm designation according to animal species hosted: Gt1-4 = Goat farms; DC1-12 = Dairy cattle farms; Sh1-5 = Sheep farms.

^2^ AL, Alava; BI, Bizkaia; TO, Toledo; ZA, Zamora; GI, Girona; NA, Navarra; CA, Cantabria; LU, Lugo; SS, Gipuzkoa.

### DNA extraction and PCR

All the samples were subjected to DNA extraction using the BioSprint 96 DNA Blood Kit (Qiagen, Hilden, Germany) following the procedure as described before [22,23]. To rule out contamination, negative controls were included during the DNA extraction process every ten (milk, vaginals swab or environmental) samples or after each placenta sample. Extraction controls and PCR negative (water) controls were subjected to PCR amplification along with the field samples. Conventional PCR [24] was used to detect the presence of *C. burnetii* DNA. After PCR confirmation, samples were analyzed by quantitative real time PCR (qPCR) in order to quantify the bacterial burden using the commercial Kit LSI Taq-Vet *Coxiella burnetii* (Laboratoire Service International, Lissieu, France) according to the manufacturer’s instructions. This is a duplex qPCR assay that targets the IS*1111* insertion element of *C. burnetii* and includes a probe targeting the housekeeping gene GAPDH used as internal amplification control (IAC) to reveal possible inhibitors. PCR was performed using an ABI 7500 FAST thermocycler (Applied Biosystems, Foster City, CA, USA).

### Multiple locus variable number tandem-repeat analysis (MLVA)

Two multicolor multiplex PCR assays were applied targeting six microsatellite markers containing either six or seven base pairs (bp) repeat units: 3 hexanucleotide repeat markers (Ms27, Ms28 and Ms34) and 3 heptanucleotide repeat markers (Ms23, Ms24 and Ms33).

Primer sequences were used as described before [13,25]. PCR was performed in a total volume of 20 μl containing 1 U of FastStart Taq DNA polymerase (Roche diagnostics, Almere, The Netherlands), 0.2 mM dNTP’s, 4 mM MgCl_2_ in 1x reaction buffer, 0.1 – 1.0 μM of amplification primers and 5 μl of DNA sample. Amplification products were analyzed on a MegaBACE 500 automated DNA analysis platform (GE Healthcare, Diegem, Belgium). Electropherograms were analyzed using Fragment Profiler 1.2 (GE Healthcare, Diegem, Belgium). DNA from the Nine Mile strain (RSA 493) was used as a reference. The number of repeats in each marker was determined by extrapolation using the sizes of the obtained fragments relative to those obtained using DNA from the Nine Mile strain. According to the *in silico* analysis, the genotype of the Nine Mile strain is 9-27-4-6-4-5 for markers Ms23-Ms24-Ms27-Ms28-Ms33-Ms34, respectively. To study the genetic similarity between the MLVA genotypes obtained in the different ruminant species the minimum spanning tree method was used.

### Multispacer sequence typing (MST)

A subset of 15 samples was selected for MST analyses according to animal species, sample source and origin. Methods and all sequences of primers have been previously detailed [16], and 8 out of the 10 spacers that exhibited higher variation (Cox2, Cox5, Cox18, Cox22, Cox37, Cox51, Cox56 and Cox61) were selected for genotyping. Each 20 μl amplification reaction contained 0.5 μM of amplification primers, 1 U of FastStart Taq DNA polymerase (Roche diagnostics, Almere, The Netherlands), 0.2 mM dNTP’s, 1.5 mM MgCl_2_ in 1x reaction buffer and 5 μl of DNA sample. After amplification, PCR products were cleaned and sequencing was performed using the forward and reverse primers. Sequence products were analyzed on a MegaBACE 500 automated DNA analysis platform (GE Healthcare, Diegem, Belgium) and using BioNumerics software (Applied Math, Sint-Martens-Latem, Belgium). The genotypes identified by MST were compared to genotypes included in the MST database containing *C. burnetii* genotypes from countries throughout Europe and from several non-European countries (http://ifr48.timone.univ-mrs.fr/MST_Coxiella/mst/).

## Results

All 45 samples were qPCR positive with cycle threshold (Ct) values below 35 and all of them were genotyped by MLVA. Eleven MLVA genotypes were identified in 35 (77.8%) of the goat, sheep and cattle specimens; partial MLVA genotypes were obtained in 6 samples (13.3%), and in 4 samples (8.9%) no MLVA profile was obtained (Table 2). In 2 of the samples (low DNA load; high Ct-value) that yielded a partial genotype (from farms DC4 and Sh2), the combination of identified alleles did not match with any of the full MLVA genotypes found, suggesting that they corresponded to different types. Three different MLVA genotypes were found in 4 goat farms; 7 MLVA genotypes were identified in 12 cattle herds; and 4 MLVA genotypes were identified in 5 sheep flocks (Table 2). Genotype S was the most abundant and present in all three ruminant species (goats, sheep and cattle), being particularly widespread in sheep (present in 3 of the 4 ovine farms sampled). Genotype T was found in goats and sheep. Multiple genotypes were identified in different samples obtained from the same farm, e.g. in farm Sh2, genotype AA was identified in samples taken from aborted ewes, and two different genotypes (Z and a partial genotype) were identified in air samples sampled during the next two reproductive seasons (Table 2).

**Table 2:** **MLVA genotyping results of *C. burnetii *strains isolated from domestic ruminants in Spain**

						**MLVA-6**					
**Farm**^**1**^	**Source**	**Origin**	**Year**	**Ct**	**MLVA**	**Ms23**	**Ms24**	**Ms27**	**Ms28**	**Ms33**	**Ms34**
Gt1	placenta	Alava	2010	8.9	S	1	11	2	3	2	3
Gt2	placenta	Bizkaia	2010	5.4	AE	4	9	3	3	3	4
Gt3	placenta	Toledo	2010	10.1	T	3	9	4	5	2	2
Gt4	placenta	Zamora	2005	11.2	S	1	11	2	3	2	3
DC1	Vaginal swab	Girona	2011	18.3	I	6	13	2	7	4	9
DC1	Vaginal swab	Girona	2011	25.5	I	6	13	2	7	4	9
DC1	Vaginal swab	Girona	2011	14.8	I	6	13	2	7	4	9
DC1	Individual milk	Girona	2011	23.9	I	6	13	2	7	4	9
DC1	Individual milk	Girona	2011	22.3	I	6	13	2	7	4	9
DC1	Aerosol	Girona	2011	30.4	-	0^3^	0	0	0	0	0
DC2	Individual milk	Bizkaia	2010	24.2	J	6	13	2	7	4	10
DC2	Individual milk	Bizkaia	2010	31.7	Mixed^4^	6	?^5^	2	?	2	?
DC2	BTM^2^	Bizkaia	2010	26.9	J	6	13	2	7	4	10
DC3	Individual milk	Bizkaia	2010	28.0	J	6	13	2	7	4	10
DC3	Individual milk	Bizkaia	2010	23.4	J	6	13	2	7	4	10
DC4	Vaginal swab	Bizkaia	2011	34.2	Partial	0	13	0	0	0	3
DC5	Individual milk	Navarra	2011	27.7	I	6	13	2	7	4	9
DC6	BTM	Cantabria	2011	25.9	AC	6	15	2	7	4	12
DC7	Individual milk	Lugo	2011	31.7	AD	6	11	2	3	4	3
DC8	BTM	Bizkaia	2009	25.2	S	1	11	2	3	2	3
DC9	BTM	Bizkaia	2009	26.1	AB	6	13	2	7	4	12
DC10	BTM	Bizkaia	2010	23.7	-	0	0	0	0	0	0
DC11	BTM	Bizkaia	2009	27.3	I	6	13	2	7	4	9
DC12	BTM	Bizkaia	2009	28.6	M	6	13	2	7	4	11
Sh1	placenta	Alava	2004	6.4	T	3	9	4	5	2	2
Sh2	Vaginal swab	Gipuzkoa	2007	10.8	AA	3	9	5	5	2	2
Sh2	Faeces	Gipuzkoa	2007	16.0	AA	3	9	5	5	2	2
Sh2	Faeces	Gipuzkoa	2007	27.0	AA	3	9	5	5	2	2
Sh2	Individual milk	Gipuzkoa	2007	28.8	AA	3	9	5	5	2	2
Sh2	Aerosol	Gipuzkoa	2008	31.6	Z	1	11	2	3	2	2
Sh2	Aerosol	Gipuzkoa	2009	30.0	Partial	9	0	5	3	0	4
Sh3	Vaginal swab	Gipuzkoa	2008	6.3	S	1	11	2	3	2	3
Sh3	Vaginal swab	Gipuzkoa	2008	10.8	S	1	11	2	3	2	3
Sh3	Vaginal swab	Gipuzkoa	2009	27.9	S	1	11	2	3	2	3
Sh3	Placenta	Gipuzkoa	2010	30.0	S	1	11	2	3	2	3
Sh3	Aerosol	Gipuzkoa	2011	32.8	S	1	11	2	3	2	3
Sh4	Vaginal swab	Gipuzkoa	2008	12.6	S	1	11	2	3	2	3
Sh4	Vaginal swab	Gipuzkoa	2008	25.0	Partial	4	11	0	3	0	0
Sh4	Aerosol	Gipuzkoa	2008	28.2	S	1	11	2	3	2	3
Sh4	Enviromental samples	Gipuzkoa	2011	32.6	S	1	11	2	3	2	3
Sh4	Enviromental samples	Gipuzkoa	2011	30.9	-	0	0	0	0	0	0
Sh5	Vaginal swab	Gipuzkoa	2008	7.1	-	0	0	0	0	0	0
Sh5	Faeces	Gipuzkoa	2008	28.5	S	1	11	2	3	2	3
Sh5	Aerosol	Gipuzkoa	2010	32.9	Partial	0	11	0	0	0	0
Sh5	Enviromental samples	Gipuzkoa	2011	29.7	Partial	0	0	0	3	0	4

^1^ Farm designation according to animal species hosted: Gt1-4 = Goat farms; DC1-12 = Dairy cattle farms; Sh1-5 = Sheep farms.

^2^ BTM = Bulk tank milk.

^3^ 0 = no results obtained.

^4^ Mixed = 2 or more genotypes.

^5^ ? = Multiple alleles were found per locus.

**Figure 1:**
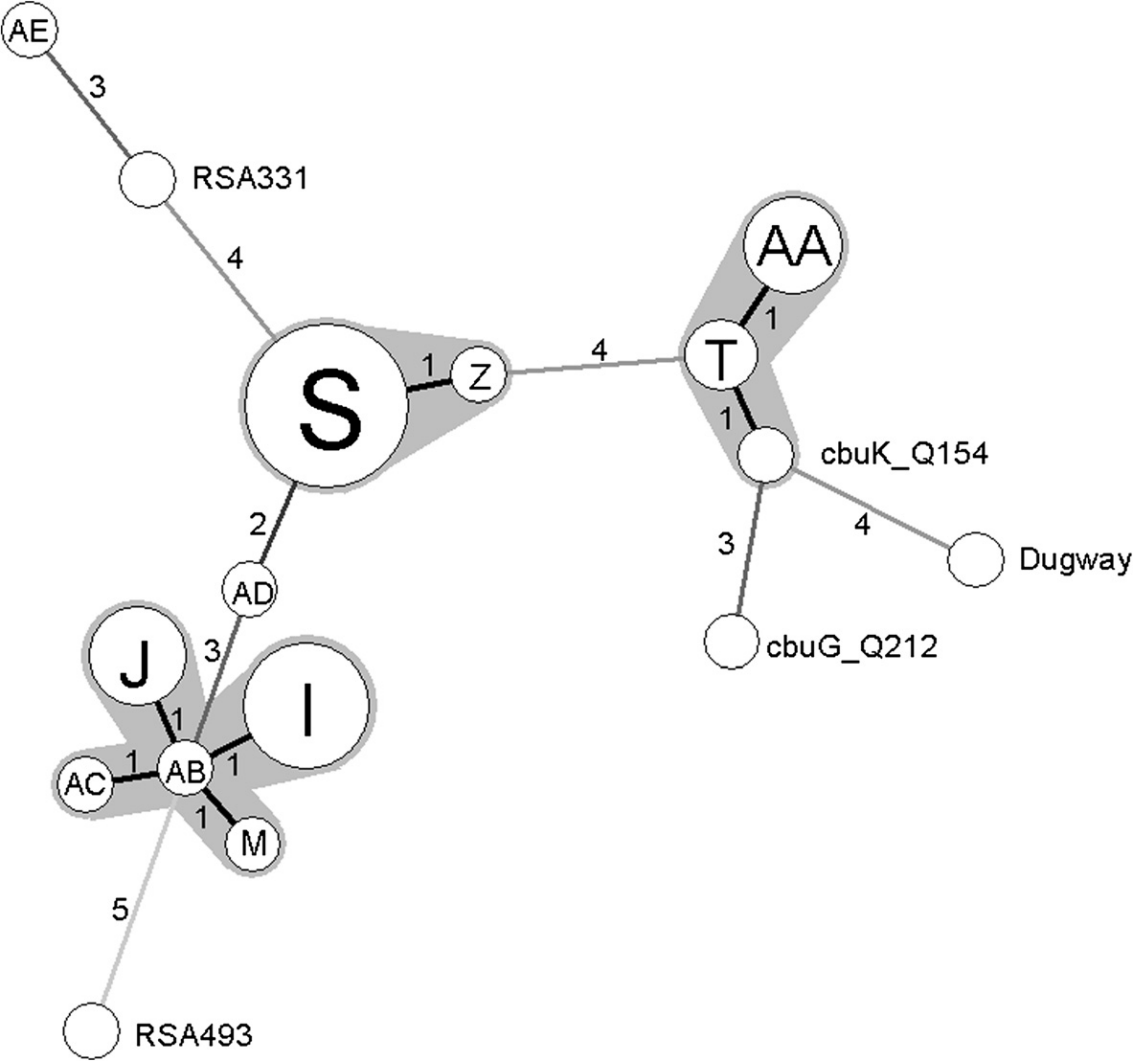
**Minimum spanning tree showing the relationship between the obtained MLVA genotypes identified in this study and five sequenced *C. burnetii *strains, i.e. Dugway (Genbank accession number CP000733), RSA331 (CP000890), Nine Mile RSA493 (AE016828), CbuG Q212 (CP001019) and CbuK Q154 (CP001020) were determined *in silico ***[13]**using the published sequences.** Each circle represents a unique genotype; the size of the circle corresponds to the number of samples with that genotype. Only full MLVA genotypes were included in this analysis. Branch labels and connecting lines correspond to the number of different markers between the genotypes. Genotypes connected by a gray background differ in only one marker from each other and may represent microvariants of one founder genotype. One cluster represents genotypes (I, J, M, AB and AC) obtained exclusively in cattle. The genotypes from cattle, goats and sheep (S, Z, AA and T) are clustered in two other groups.

Figure 1 shows the relationships between all identified genotypes from goats, sheep and cattle in Spain. Clustering of the MLVA genotypes using the minimum spanning tree method showed a high diversity between the strains. Totally, three different clusters were defined. The genotypes in cluster one (I, J, M, AB and AC) were all obtained from cattle and are interconnected by repeated number changes in one of the six markers. In addition, one cattle isolate (genotype AD) differed in three markers with the cattle strains of cluster one, and also differed in at least two markers with the genotypes in cluster two (S and Z). MLVA genotype AA of cluster three, detected in sheep, differed in only one allele from genotype T found in a goat and a sheep sample.

MST analysis of the 15 samples selected (4 goats, 4 sheep, 7 cattle) revealed 4 different MST genotypes 8, 13, 18 and 20 (Table 3). MST13 was identified in all three ruminant species (goats, sheep and cattle); MST20 was detected more than once, but always in cattle; MST18 was only identified once in a goat placenta; and, MST8 was identified in goat. In addition, some partial MST genotypes found in sheep (samples from farms Sh1 and Sh2) might also correspond to MST8. Correspondence between genotyping results by MLVA and MST are shown in Table 3. MLVA genotypes belonging to the same MLVA cluster all yielded the same MST genotype.

**Table 3:** **MLVA and MST genotypes of *C. burnetii *strains isolated from domestic ruminants in Spain**

							**MST**							
**Farm**	**Source**	**Origin**	**Year**	**Ct**	**MLVA**	**MST**	**COX2**	**COX5**	**COX18**	**COX22**	**COX37**	**COX51**	**COX56**	**COX61**
Gt1	placenta	Alava	2010	8.9	S	13	3	5	1	5	4	5	5	2
Gt2	placenta	Bizkaia	2010	5.4	AE	18	3	8	1	3	4	7	−^1^	3
Gt3	placenta	Toledo	2010	10.1	T	8	5	4	2	1	5	3	3	4
Gt4	placenta	Zamora	2005	11.2	S	13	3	5	1	5	4	5	5	2
DC1	Individual milk	Girona	2011	23.9	I	20	3	2	6	5	4	4	10	5
DC2	Individual milk	Bizkaia	2010	24.2	J	20	3	2	6	5	4	4	10	5
DC2	BTM	Bizkaia	2010	26.9	J	20	3	2	6	5	4	4	10	5
DC5	Individual milk	Navarra	2011	27.7	I	20*	3	2	6	5	4	4	-	5
DC6	BTM	Cantabria	2011	25.9	AC	20	3	2	6	5	4	4	10	5
DC7	Individual milk	Lugo	2011	31.7	AD	13	3	5	1	5	4	5	5	2
DC8	BTM	Bizkaia	2009	25.2	S	13*	3	5	1	5	4	5	-	2
Sh1	placenta	Alava	2004	6.4	T	8*	5	4	2	1	5	-	3	4
Sh2	Faeces	Gipuzkoa	2007	16.0	AA	8*	5	4	2	1	5	3	-	4
Sh3	Vaginal swab	Gipuzkoa	2009	27.9	S	13	3	5	1	5	4	5	5	2
Sh4	Vaginal swab	Gipuzkoa	2008	25.0	Partial	13	3	5	1	5	4	5	5	2

^1^ = no result obtained in the corresponding spacer, giving an estimated MST profile marked with an asterisk (*).

## Discussion

Molecular methods are used to characterize strains and to determine relationships between isolates causing disease. In the case of Q fever, MLVA and MST techniques have been incorporated for genotyping of *C. burnetii* strains since both techniques can be performed directly on clinical and environmental samples without previous cultivation of bacteria [11,16]. In the current study MLVA typing has been performed based on 6 loci on 45 *C. burnetii*-positive samples to study the genetic background of this bacterium in domestic ruminants in Spain.

MLVA typing revealed a substantial genetic diversity among *C. burnetii* from domestic ruminants in Northern Spain as shown in the minimum spanning tree, with 11 distinct genotypes being identified. None of the MLVA profiles found here were similar to the profiles identified in the Q fever outbreak episodes in The Netherlands [13] or Poland [26]. The MLVA genotypes (I, J, M, S and T) described in the current study have been found before, indicating a wide dissemination of the described MLVA genotypes throughout Europe. MLVA genotypes I, J and M have been found in cattle milk from France, Netherlands, Portugal, Spain and Switzerland [14], and have also been incidentally found in 8 human clinical samples (placenta and heart valve) from France, according to an in-house database containing 61 different *C. burnetii* MLVA genotypes from 231 human, caprine, ovine and cattle clinical samples and cows milk obtained from Canada, France, Germany, The Netherlands, Portugal, Qatar, Russia, Saudi Arabia, Slovak Republic, Spain, Switzerland, United Kingdom and USA. Moreover, MLVA genotypes S and T have also been incidentally found in 8 human clinical samples (blood and valve) from France and Portugal and in 6 ruminant samples (goat and sheep) from Portugal [27].

In addition, 6 new MLVA profiles were identified (Z and AA in sheep, AE in goats, and AB, AC, and AD in cattle) which so far have not been detected in human or animal samples. However, some of these new genotypes differ in only one marker from other previously defined and may represent microvariants of the founder genotype.

Interestingly, variations in MLVA genotypes were observed throughout consecutive reproductive seasons in some sheep farms. This was the case on Farm Sh2, where genotypes detected in air samples were different from those detected in aborted ewes. This also happened in farms Sh4 and Sh5, where partial genotypes in environmental or animal samples were different. Typing data provided important epidemiological information about the sources of infection, and explained previous observations when *C. burnetii* appeared in environmental surfaces while no animal shedders were present in the sheep flocks [28].

Since several *C. burnetii* genotypes can be present on a farm, BTM samples might be contaminated with several different genotypes. However, apart from one sample (from farm DC10) that could not be typed, clean chromatograms were obtained by MST in all BTM samples tested in this study, suggesting the presence of only one genotype per BTM sample. This was also supported by the MLVA results.

Looking at MLVA genotyping results on individual milk samples, apart from one sample from farm DC2, only one genotype was detected per individual milk sample and per farm, as shown in farms DC1 and DC3. In the sample from farm DC2 (Bizkaia region), more than one allele per locus was observed, suggesting the presence of at least two or more different MLVA genotypes. In addition, the presence of highly similar *C. burnetii* genotypes (I, J, M, AB and AC) in cattle milk may indicate a widespread dissemination of a specific cattle-adapted strain, as previously reported [14].

MLVA typing has shown to be less laborious and more discriminatory than MST [15]. However, MST has the advantage of using standardized nomenclature, and having databases that allow easy comparison of results between laboratories and studies. It is interesting that the MST genotype involved in the human Q fever outbreak in The Netherlands (MST33), linked to goats and sheep and found also in Germany and France [17], was not detected in the present study. However, the most common genotype (MST13), which was identified in the three ruminant species in Spain, had been identified before in human Q fever cases in France, and recently in Portugal [27]. Other MST genotypes detected in this study, had also been previously reported. MST8, detected here in goats and probably present in sheep (partial profile), has been found before in human samples and in one ovine sample from Spain, France and USA, and in human Q fever chronic cases from Portugal [27]. MST18, found only on 1 goat farm was isolated before from human and animal (sheep and goats) clinical samples in France, Italy, Romania, Greece, Slovak Republic and Germany according to the MST database (http://ifr48.timone.univ-mrs.fr/MST_Coxiella/mst/). Finally, MST20, found here in cattle, had been identified in animal and human clinical samples from France, Germany, Netherlands and USA [16,17]. Human isolates need to be genotyped with the same techniques used here on animal samples to identify the most important animal source for human Q fever infection in this Spanish region. The only genotyping study carried out in Spain, used PCR and RLB hybridization to determine the presence/absence of 8 ORFs in order to compare *C. burnetii* isolates from domestic ruminants (n = 29) and human cases (n = 24). The authors identified some related genomic groups in *C. burnetii* isolated from humans, sheep and goats, but not from cattle [21]. This is in agreement with the results obtained in The Netherlands, where prevalence of *C. burnetii* DNA in dairy cattle is high [29] but MLVA and MST genotypes detected in cattle are different from those involved in the human Q fever outbreak [12-14,17].

## Conclusions

Understanding the distribution of *C. burnetii* genotypes present in a region is critical to identify the major sources of infection, and implement efficient farm-based control measures to reduce human exposure to the pathogen. However, it is necessary to harmonize genotyping techniques to be used in *Coxiella* epidemiological studies, so that results can be exchanged and readily comparable among different laboratories and studies. Likewise, a common website where all typing data can be submitted and easily accessed is necessary for timely identification of new strains.

## Abbreviations

MLVA: Multiple locus variable number tandem repeats analysis; MST: Multispacer sequence typing; qPCR: Real-time PCR; IAC: Internal amplification control; Ct: Cycle threshold.

## Competing interests

Authors declare that there are no financial competing interests.

## Authors’ contributions

JA and JT were responsible for laboratorial analyses and assisted with interpretation of data; AP provided samples and made DNA extractions; JT and AH assisted with discussion of results and writing the manuscript; ALG coordinated sample selection and wrote the manuscript; MNF and CK supervised laboratory work and critically revised the manuscript. All authors revised the manuscript and approved it in its final version.
